# Transcriptome and Proteome Analyses Reveal Stage-Specific DNA Damage Response in Embryos of Sturgeon (*Acipenser ruthenus*)

**DOI:** 10.3390/ijms23126392

**Published:** 2022-06-07

**Authors:** Ievgeniia Gazo, Ravindra Naraine, Ievgen Lebeda, Aleš Tomčala, Mariola Dietrich, Roman Franěk, Martin Pšenička, Radek Šindelka

**Affiliations:** 1 South Bohemian Research Center of Aquaculture and Biodiversity of Hydrocenoses, Faculty of Fisheries and Protection of Waters, University of South Bohemia in České Budějovice, Zátiší 728/II, 389 25 Vodňany, Czech Republic; ilebeda@frov.jcu.cz (I.L.); franek@frov.jcu.cz (R.F.); psenicka@frov.jcu.cz (M.P.); 2 Laboratory of Gene Expression, Institute of Biotechnology—Biocev, Academy of Science of Czech Republic, 252 50 Vestec, Czech Republic; ravindra.naraine@ibt.cas.cz (R.N.); radek.sindelka@ibt.cas.cz (R.Š.); 3 Institute of Aquaculture and Protection of Waters, Faculty of Fisheries and Protection of Waters, University of South Bohemia in České Budějovice, Husova tř. 458/102, 370 05 České Budějovice, Czech Republic; tomcala@frov.jcu.cz; 4 Department of Gametes and Embryo Biology, Institute of Animal Reproduction and Food Research, Polish Academy of Sciences, Tuwima 10, 10-748 Olsztyn, Poland; m.dietrich@pan.olsztyn.pl

**Keywords:** fish, sturgeon, embryo, DNA damage response, omics

## Abstract

DNA damage during early life stages may have a negative effect on embryo development, inducing mortality and malformations that have long-lasting effects during adult life. Therefore, in the current study, we analyzed the effect of DNA damage induced by genotoxicants (camptothecin (CPT) and olaparib) at different stages of embryo development. The survival, DNA fragmentation, transcriptome, and proteome of the endangered sturgeon *Acipenser ruthenus* were analyzed. Sturgeons are non-model fish species that can provide new insights into the DNA damage response and embryo development. The transcriptomic and proteomic patterns changed significantly after exposure to genotoxicants in a stage-dependent manner. The results of this study indicate a correlation between phenotype formation and changes in transcriptomic and proteomic profiles. CPT and olaparib downregulated oxidative phosphorylation and metabolic pathways, and upregulated pathways involved in nucleotide excision repair, base excision repair, and homologous recombination. We observed the upregulated expression of zona pellucida sperm-binding proteins in all treatment groups, as well as the upregulation of several glycolytic enzymes. The analysis of gene expression revealed several markers of DNA damage response and adaptive stress response, which could be applied in toxicological studies on fish embryos. This study is the first complex analysis of the DNA damage response in endangered sturgeons.

## 1. Introduction

Genotoxicity is the property of chemical agents to induce DNA damage, modifications, rearrangements, and mutations. The issue of genotoxicity has become increasingly important as numerous genotoxic compounds have been found in the environment [1]. Previous studies have shown that genotoxic compounds may have a long-term impact on organisms [2,3], particularly on developing embryos [4]. Embryogenesis is sensitive to DNA damage as the cell cycle is short and DNA replication is fast [5]. Furthermore, most vertebrate species lack functional cell-cycle control checkpoints at the early stages of development [6]. Subsequently, if embryos are exposed to genotoxicants before checkpoint activation, DNA synthesis and cell division continue in the presence of damaged DNA. In fish, after the midblastula transition (MBT) checkpoints become activated, they can block developing embryos until DNA is repaired or apoptosis is initiated [7]. Thus, embryonic development and the cell cycle are closely associated with DNA repair.

The main DNA damage response (DDR) pathways, which are highly conserved between organisms [8], include nucleotide excision repair (NER), base excision repair (BER), DNA mismatch repair (MMR), homologous recombination (HR), and nonhomologous end joining (NHEJ) [9]. Nevertheless, the activity of these pathways during embryo development and their ability to mitigate DNA damage at different stages is still not fully described [10], particularly in non-model species.

In the current study, the focus was made on the embryonic response to DNA damage in sterlet (*Acipenser ruthenus*). The sterlet belongs to the family of sturgeons (Acipenserids), a group of ray-finned fish, which likely evolved approximately 200 million years ago [11]. They have been classified as vulnerable fishes by international organizations (available online: https://www.iucnredlist.org/ (accessed on 31 May 2022); [12]). Sturgeons are important aquaculture species known for their meat and caviar production. They represent an attractive model for studying DDR owing to their multiple rounds of lineage-specific whole-genome duplication [13]. Furthermore, sturgeons have extraordinary genomic plasticity, as demonstrated through intraspecific hybridization of individuals with different chromosome numbers [14], events of spontaneous polyploidization [15,16], and artificial ploidy manipulation that yields the highest documented chromosome number in vertebrates [17]. According to the authors of [18], genome size is linearly related to the number of DNA repair genes and proteins. Taking all this into account, it is possible to speculate that the sturgeon’s genome plasticity would require a highly effective DDR.

Several studies have already investigated the complex effects of water pollution on the transcriptome and/or proteome of model species, such as zebrafish (*Danio rerio*) [19,20]. Such advanced “omics” approaches can decipher the mode of action of the compound [19] and help to understand the organism’s response to certain types of toxicity. One of the most popular “omics” techniques for the determination of adverse effects is transcriptomics (analysis of RNA transcript abundance). There are several advantages to transcriptomics, such as the low quantity of the biological material needed for analysis, high sensitivity, and robustness [21]. However, the key regulators of the cellular pathways are proteins, which can undergo post-translational modifications and degradation; thus, the levels of functionally available proteins may be independent of transcription [22]. However, 2D gel protein electrophoresis combined with mass spectrometry (MS) has limited sensitivity and requires a large quantity of biological material.

Surprisingly, very few studies have performed complex analyses of the DDR in aquatic organisms, even though DNA damage is commonly used as a biomarker of exposure [23,24]. Further, it is important to establish the link between phenotype formation and changes in “omics” profiles. It is possible to hypothesize that treatment with the same concentration of a genotoxicant at different stages could induce different phenotypes. Therefore, by using stage specificity of DDR and different types of DNA damaging agents, it could be possible to establish molecular markers of adverse effects. In the present study the effect of two genotoxic compounds, camptothecin (CPT) and olaparib (Ola), on sterlet embryo development was analyzed at different stages. Camptothecin is an inhibitor of topoisomerase I (Top1), an enzyme that regulates DNA topology [25]. CPT stabilizes Top1–DNA complexes leading to their conversion into DNA single- and double-strand breaks (SSB and DSB). Olaparib is an inhibitor of poly (ADP-ribose) polymerase-1 (PARP-1), a protein that plays a crucial role in DNA damage sensing and repair [26]. Both chemicals are commonly used in studies on DNA damage and repair [7,27,28]. To understand the DDR in fish embryos, the effect of CPT and Ola on embryo morphology, DNA fragmentation, the transcriptome, and the proteome was analyzed at different stages of embryo development.

## 2. Results

### 2.1. Effect of Genotoxicants on Embryo DNA Integrity and Phenotype Formation

**Figure 1:**
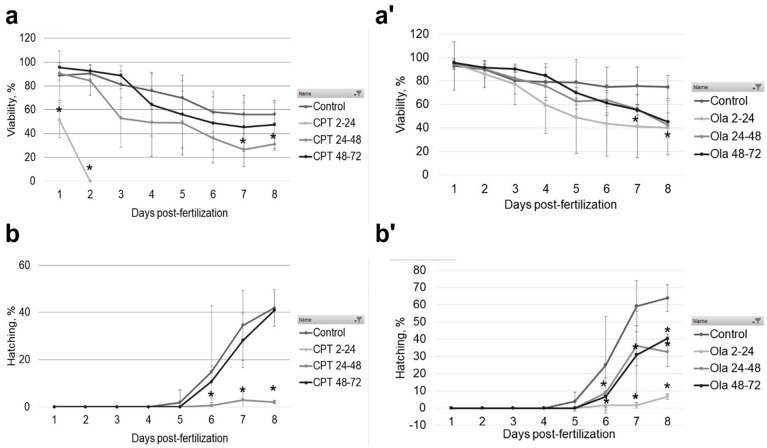
Sterlet (*A. ruthenus*) embryo survival rate after camptothecin (CPT) (**a**) and olaparib (Ola) (**a’**) exposure. Embryos were exposed to CPT and Ola at 2–24, 24–48, and 48–72 hpf. Embryo viability was monitored daily for 8 days. The hatching rate after CPT (**b**) and Ola (**b’**) exposure. Results represent the mean of six independent experiments. Error bars represent the standard error of the mean. Asterisks indicate significant differences compared with the control at the same time point (* *p* < 0.05, Kruskal-Wallis test).

**Figure 2:**
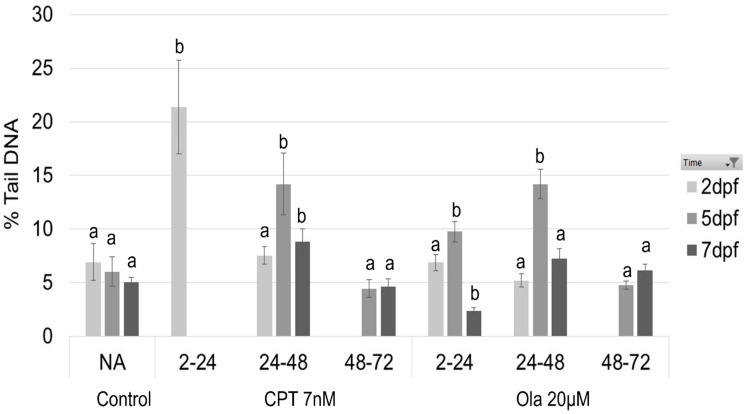
DNA damage analyzed by the comet assay as %TailDNA. Sterlet (*A. ruthenus*) embryos were exposed to CPT and Ola at 2–24, 24–48, and 48–72 hpf. Percentage DNA in the comet tail was analyzed at 2, 5, and 7 dpf. Letters indicate a significant difference between the treatment group and the control at the same dpf (ANOVA, *p* < 0.05).

Sterlet (*A. ruthenus*) embryos were exposed to CPT and Ola at different stages of embryonic development. The results of CPT exposure on sturgeon embryo development (phenotype formation and DNA damage) have been described in detail in our previous study [10]. Briefly, 7 nM CPT was lethal for sterlet embryos exposed at the early stage (2–24 hpf), a mortality rate of up to 70% was induced when embryos were exposed during the gastrula stage (24–48 hpf), with a slight effect on viability following exposure during the neurula stage (48–72 hpf) (Figure 1a). The hatching rate was unaffected when embryos were exposed to CPT during the neurula stage, whereas embryos exposed to CPT during gastrulation showed a low hatching rate (2%) (Figure 1b). The level of DNA fragmentation reached 21.3% TailDNA in the CPT 2–24 group (Figure 2). The embryos in the CPT 24–48 group showed the highest level of DNA fragmentation at 5 dpf (14.2% TailDNA), which decreased by 7 dpf (Figure 2). The sterlet embryos in the CPT 48–72 group showed no increased DNA fragmentation compared with the control. The images of the phenotypes observed following CPT exposure are presented elsewhere [10].

**Figure 3:**
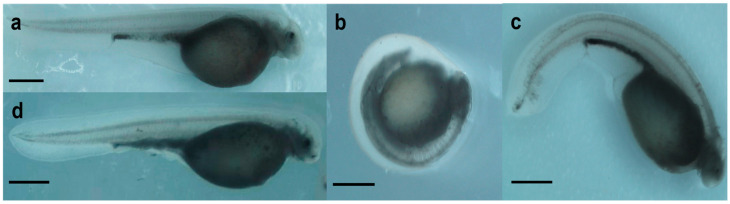
Sterlet (*A. ruthenus*) embryo phenotypes observed at 8 dpf following exposure to olaparib (Ola) at 2–24, 24–48, and 48–72 hpf. (**a**) Control; (**b**) Ola 2–24; (**c**) Ola 24–48; (**d**) Ola 48–72. Images are representative of embryo culture. Scale bars = 1 mm.

Exposure to 20 µM Ola did not lead to 100% mortality, even at early stages (2–24 hpf) (Figure 1a’). Nevertheless, the viability of the embryos decreased significantly compared with the control (Figure 1a’). Further, the hatching rate was as low as 6.8% in embryos exposed to Ola at 2–24 hpf (Figure 1b’). When embryos were exposed to a PARP-1 inhibitor at the later stages, the hatching rate at 8 dpf reached 32.8% for Ola 24–48 hpf and 40.4% for Ola 48–72 hpf (Figure 1b’). Similarly, DNA fragmentation at 5 dpf was significantly higher for Ola 2–24 hpf and Ola 24–48 hpf compared with the control (Figure 2). However, the group Ola 48–72 did not show a significant increase in DNA fragmentation. Furthermore, no phenotype formation was observed in this group, unlike in embryos exposed at earlier stages (Figure 3). Embryos exposed to Ola at the early stages showed skeletal malformations and a phenotype similar to developmental delay (Figure 3b,c).

For RNA sequencing and proteomic analysis, we selected the embryos from the CPT 24–48, CPT 48–72, Ola 2–24, and Ola 24–48 hpf groups. CPT exposure in the earlier stage was lethal and thus no embryos survived until hatching. Furthermore, the CPT 24–48 and CPT 48–72 groups showed very different patterns of DNA damage and different phenotypes, which made them of interest for further analysis. Similarly, the embryos exposed to Ola at 2–24 and 24–48 hpf showed different levels of DNA fragmentation and phenotype formation. Embryos at the hatching stage were selected for further analysis as, at this stage, differences in phenotype formation between treatments were the most prominent. In addition, our aim was to determine the DDR pathways that led to DNA repair observed at 7 dpf in all chosen groups (Figure 2).

### 2.2. Main Changes in Transcriptomic Patterns in Sturgeon Embryos after DNA Damage

**Figure 4:**
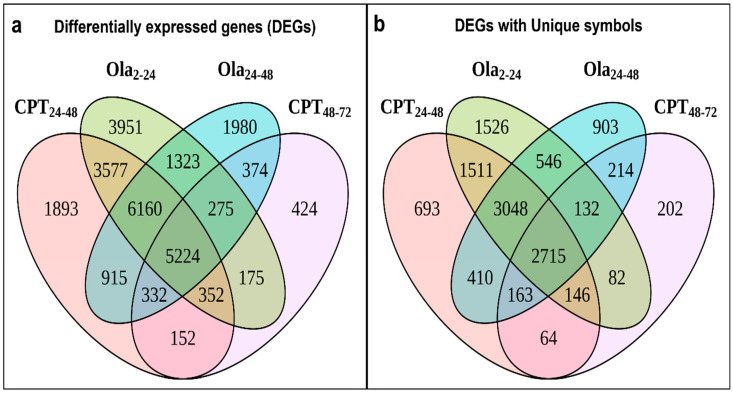
Overlap in significantly (padj < 0.1) differentially expressed genes (DEGs) of sterlet (*A. ruthenus*) between treatments (**a**). Only DEGs with unique gene symbols (**b**).

The transcriptome of sterlet embryos (N = 3–4 for each group) was compared between the control and the CPT 24–48, CPT 48–72, Ola 2–24, and Ola 24–48 groups at 7 dpf. It has been previously shown that the total sterlet genome comprises 120 chromosomes, about 47,500 protein-coding genes, and 1.8 billion base pairs [13]. A total of 45,030 transcripts were identified in sturgeon embryos, with 18,605 differentially expressed genes (DEGs) in CPT 24–48, 7308 DEGs in CPT 48–72, 21,037 DEGs in Ola 2–24, and 16,583 DEGs in Ola 24–48 compared with the control embryos (Figure 4, Supplementary Table S1). Interestingly, the comparison of the results among different treatment groups showed a high number of overlapping DEGs in CPT 24–48 and Ola 2–24 (approximately 56% of DEGs, 15,313 DEGs in total) compared with 22% overlapping DEGs in CPT 24–48 and CPT 48–72. The groups of Ola 2–24 and Ola 24–48 shared a total of 48% of similarly expressed genes and Ola 24–48 and CPT 48–72 shared 23% DEGs. Overall, 19% overlapping DEGs were detected between all treatments, and 42% overlapping DEGs were observed among the CPT 24–48, Ola 2–24, and Ola 24–48 groups (Figure 4).

The DEGs that were detected with a lower mRNA abundance in the treatment groups compared with the control (log2 fold-change < 0) were referred to as “downregulated”, and those that showed a higher abundance in the treatment compared with the control (log2 fold-change > 0) were defined as “upregulated”. The enrichment of gene ontologies associated with biological processes was determined for the downregulated and upregulated genes. There were very few downregulated GO terms associated with biological processes in the CPT 48–72 group (Supplementary Figure S1, Supplementary Table S1). The CPT 24–48 and Ola 2–24 groups shared a similar distribution of enriched GO terms. Ola 24–48 had specific downregulation of terms associated with the electron transport chain and peptide biosynthesis. There was also specific downregulation of terms associated with development (e.g., circulatory system), transport, and apoptotic cell clearance in the CPT 24–48 and Ola 2–24 groups.

**Figure 5:**
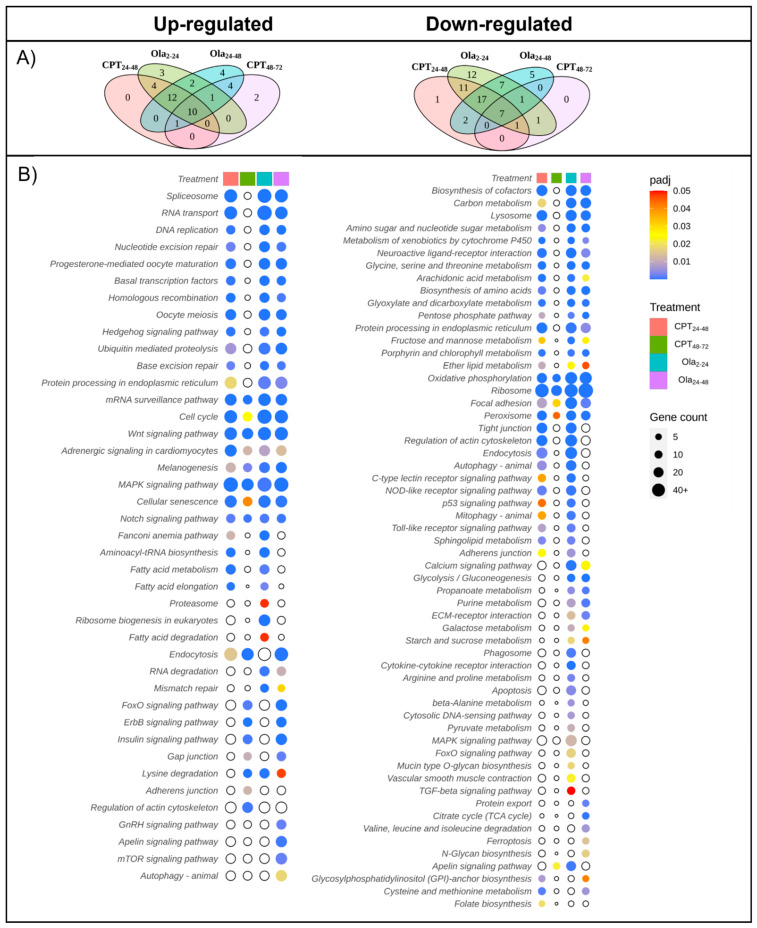
Enriched KEGG pathways in the treated versus the control embryos of sterlet (*A. ruthenus*). (**A**) Overlap in the enriched KEGG pathways between treatments; (**B**) visualization of the level of significance and number of representative DEGs found in each pathway for each treatment group. The color scale represents adjusted *p*-values less than 0.05, with empty circles showing those greater than 0.05. The size of the circle represents the number of significantly differentially expressed genes that are part of the given KEGG pathway.

The KEGG pathway enrichment analysis showed the downregulation of oxidative phosphorylation in all treatment groups (Figure 5). Only the CPT 24–48 and Ola 2–24 groups had the downregulation of the p53 signaling pathway and autophagy pathways, whereas the autophagy pathway was, in contrast, upregulated in Ola 24–48. There was downregulation of pathways associated with sugar metabolism, protein processing, and the biosynthesis of amino acids in all treatment conditions, except CPT 48–72.

In comparison with the control embryos, all treatment groups also shared several upregulated GO main terms (Supplementary Figure S2, Supplementary Table S1). These were related to biological processes, such as the increase in the regulation of cellular and metabolic processes, RNA biosynthesis, and macromolecules. There were also upregulated terms associated with the organization of cellular components, chromatin, and the cytoskeleton in all treatment groups. Upregulation was also observed for GO terms associated with the cell cycle and cell cycle phase transition, but these were only seen in the Ola 2–24 and Ola 24–48 groups, a few in CPT 24–48, and were completely lacking in CPT 48–72.

The following KEGG pathways were upregulated in all treatment groups: adrenergic signaling in cardiomyocytes, cell cycle, Wnt signaling pathway, cellular senescence pathway, MAPK signaling pathway, melanogenesis, Notch signaling pathway, and mRNA surveillance pathway (Figure 5). RNA degradation and mismatch repair were only observed in the Ola-treatment groups. The FoxO and ErbB signaling pathways were only upregulated in the CPT 48–72 and Ola 24–48 groups.

Enrichment of GO terms was analyzed for the downregulated DEGs in the “developmental processes” category (Supplementary Figure S3). The results of this analysis revealed very limited downregulation of GO terms. The upregulated GO terms included anatomical structure development, animal organ development, brain development, eye development, cell development, head development, and sensory system development in all treatment groups (Supplementary Figure S3). The upregulation of GO terms related to embryonic development ending in birth or egg hatching, chordate embryonic development, and embryo development was detected only in the CPT 24–48, Ola 2–24, and Ola 24–48 treatment groups.

### 2.3. Differentially Expressed Genes Associated with Cell Cycle and DNA Repair Pathways

The upregulation of the KEGG pathways involved in NER, BER, and HR was observed for CPT 24–48, Ola 2–24, and Ola 24–48 groups. The upregulation of mismatch repair was observed only in Ola 2–24 and Ola 24–48 (Figure 5). The upregulation of GO terms related to DNA repair and response to stress was detected only in the CPT 24–48 and Ola-treatment groups (Supplementary Figure S2).

**Figure 6:**
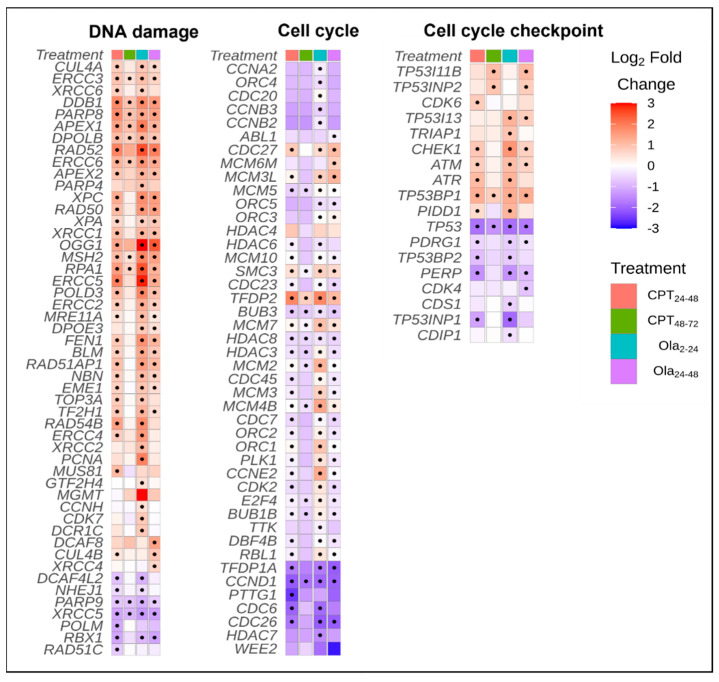
Genes associated with DNA damage, cell cycle, and cell cycle checkpoint control that were significantly (padj < 0.1) differentially expressed in at least one of the treated samples relative to the control in sterlet (*A. ruthenus*). The color scale represents the log2 fold-change between the treatment and the control and is capped at a maximum of 3 and a minimum of −3. A black dot represents the genes that were differentially expressed.

We have compared the changes in the expression of genes associated with the cell cycle, checkpoints, and DNA repair (Figure 6). The results of this analysis showed that numerous cell cycle genes were significantly upregulated in the Ola 2–24, Ola 24–48, and CPT 24–48 groups (Figure 6, Supplementary Figure S4). However, the largest fold-change in upregulation was observed in Ola 2–24. All treatment groups showed significant upregulation of *tfdp2* and downregulation of *ccnd1*. In general, CPT 24–48 and Ola resulted in similar trends in cell cycle gene expression.

Genes associated with cell cycle checkpoint were differently regulated by exposure to genotoxicants (Figure 6). Ola 2–24, Ola 24–48, and CPT 24–48 showed a similar number of DEGs and relatively similar fold changes. Gene *tp53bp1* was upregulated, and *tp53* was significantly downregulated in all treatment groups. Most of the checkpoint regulating kinases, such as *chek1*, *atr*, and *atm* were upregulated in Ola 2–24, Ola 24–48, and CPT 24–48, but not CPT 48–72.

Among the genes associated with DNA repair, *ddb1, parp8, apex1, rad52,* and *ogg1* were upregulated in all treatments, whereas *xrcc5* and *parp9* were downregulated (Figure 6). Sterlet embryos from Ola 2–24, Ola 24–48, and CPT 24–48 groups showed increased expression of *xpc*, *rad50*, *xpa*, *xrcc1*, *msh2*, *rpa1*, *ercc5*, *pold3*, *ercc2*, *fen1*, *blm*, *rad51ap1*, *nbn*, and *eme1*. In contrast, the expression of genes associated with DNA repair was only slightly increased in the CPT 48–72 group. Mapping the KEGG pathways of BER (hsa03410; Supplementary Figure S5) showed that most of the BER genes were upregulated in Ola 2–24 and Ola 24–48 groups, but unaffected in the CPT 48–72 group. In the MMR pathway (has03430; Supplementary Figure S6) most of the genes were upregulated in Ola 2–24 and Ola 24–48 groups. The NER pathway (hsa03420; Supplementary Figure S7) and HR pathway (hsa03440; Supplementary Figure S8) were upregulated in the Ola 2–24, Ola 24–48, and CPT 24–48 groups.

### 2.4. Protein Profiles in Sturgeon Embryos following DNA Damage

The 2D-PAGE protein patterns were compared between the control embryos and embryos exposed to genotoxicants (N = 3 for each group) (Supplementary Figures S9 and S10). The results of the mass spectrometric analysis of differently abundant proteins (DAPs) are presented in Supplementary Tables S2 and S3. The 25 DAPs were identified following exposure to CPT. Interestingly, from these proteins, only two followed the same trend as mRNA levels, namely cofilin-2-like (upregulated relative to control) and translationally controlled tumor protein homolog (downregulated relative to control). The most upregulated group of proteins in both CPT-treated groups (CPT 24–48 and CPT 48–72) were zona pellucida sperm-binding proteins (Supplementary Table S2). Several proteins associated with metabolic pathways were downregulated in CPT-treated embryos: putative aminopeptidase W07G4.4, alpha-enolase isoform X2, and creatine kinase M-type. This result agreed with the results of RNA sequencing from the CPT 24–48, where the DEGs associated with metabolic pathways were downregulated (Figure 5, Table 1).

The results obtained in the current study are summarized in Table 1. Our data indicate that treatment with Ola upregulated proteins is associated with KEGG metabolic pathways and peroxisome. In contrast, treatment with CPT downregulated cytoskeletal proteins (Table 1).

Correlation between viability, hatching rate, DNA fragmentation, number of DEGs, and the number of DAPs was analyzed as well (Table 2). According to Spearman’s test, there was a strong positive correlation between viability and hatching rate. Furthermore, a significant negative correlation was observed between viability and the number of DEGs, viability, and DAPs, as well as hatching and DEGs.

## 3. Discussion

The current study aimed to describe the DDR in sturgeon embryos induced by exposure to genotoxicants. Considering the endangered status of many sturgeon species and their importance to aquaculture, studies on sturgeon embryo development are highly demanded. Furthermore, the results of our research indicate that sturgeon embryos are most sensitive to stress in the early stages of development. Following neurulation, the embryos acquire mechanisms to cope with chemical exposure and/or DNA damage. This information could be helpful to aquaculture, where sturgeon embryos are produced and reared in large amounts in vitro.

It has been previously shown that CPT exposure led to different levels of DNA damage and different malformation rates, depending on the stage of exposure [10]. Similarly, Ola exposure in the early stages of development led to decreased survival and hatching rate, whereas exposure after neurulation did not have a significant effect on sturgeon embryos. In contrast to CPT, Ola exposure at early stages (2–24 hpf) did not lead to total mortality, and embryos developed up to 7 dpf. Nevertheless, these embryos failed to hatch, with a high rate of malformations, indicating that the proper functioning of PARP-1 is required for embryo development. This agreed with previous studies on zebrafish embryos that showed how the inhibition of PARP-1 could lead to increased DNA damage, cell death, and problems with development [26,28,29].

In general, gastrulation is a critical phase for fish embryos, as, at this point, cells acquire the ability to undergo apoptosis [7]. When fish embryos were treated with genotoxicants after gastrulation, they showed a higher rate of survival and hatching than the group treated before gastrulation ([7,30]; current study). There are several possible hypotheses to explain this gradual increase in the resistance of post-MBT embryos. First, it is possible to suggest that the activation of cell cycle checkpoints and apoptosis after the MBT transition allows embryos to effectively repair DNA or remove damaged cells [7]. On the other hand, it is known that, upon gastrulation, fish embryos develop an epidermis that acts as the major barrier to harmful molecules [31]. The increased expression of ATP-binding cassette transporters in the epidermis could result in higher resistance of embryos after gastrulation [32]. This is supported by the report of [26], who observed the decreased uptake of the genotoxicant doxorubicin in post-MBT zebrafish embryos compared with the early stages of development. Further studies are needed to understand the kinetics of genotoxic compounds in fish embryos at different stages of development.

The changes in the transcriptome were compared between control sturgeon embryos and embryos treated with CPT at 24–48 and 48–72 hpf and with Ola at 2–24 and 24–48 hpf. The results of our study indicate that DNA damage induced by CPT and Ola can be partially repaired. Interestingly, the results of RNA sequencing indicated that CPT 24–48 and Ola 2–24 shared more DEGs than CPT 24–48 and CPT 48–72. Similarly, the CPT 24–48 and Ola 2–24 groups had a low hatching rate, increased malformation rate, and DNA damage. These results suggest that there is a general mechanism of DDR that depends on the stage of embryo development and the level of DNA damage. Thus, both treatments altered metabolism pathways, cell proliferation, the cell cycle, and the developmental processes. This result shows that phenotypic changes in fish embryos induced by genotoxic treatments were associated with changes in gene expression. This agreed with the report of Marx-Stoelting et al. [21], who suggested that the adverse effects of chemicals should be defined based on impairment of functional capacity and pathology formation, rather than only alterations at the transcriptomic level. Thus, it is possible to draw a correlation between embryo phenotypes and “omics” data in the case of genotoxic treatments applied in the early stages of embryo development (Table 2).

In contrast, the CPT 48–72 group developed no phenotype, but several pathways, such as developmental pathways, oxidative phosphorylation, metabolism, and the regulation of the cytoskeleton, were significantly altered at the transcriptome and proteome levels. In this case, it was difficult to distinguish between the adverse effect of genotoxicity and adaptive response. However, we observed significant upregulation of developmental processes and nervous system development in this treatment. Previous studies indicated that early life stress may have large effects on the developing brain and nervous system in vertebrates [33,34]. Further studies are needed to understand if exposure to genotoxicants after neurulation may affect sturgeons later in life and subsequently induce adverse effects.

The analysis of gene expression associated with cell cycle and DNA repair showed that several genes were upregulated in all treatment groups, such as *tfdp2*, *tp53bp1*, *ddb1*, *parp8*, *apex1*, *rad52*, and *ogg1*, whereas *ccnd1*, *tp53*, *parp9*, and *xrcc5* were downregulated. Thus, the cell cycle, p53 signaling, and some DDR pathways were affected by all test conditions. Interestingly, we observed the downregulation of *tp53* and *ccnd1* expression in most of the treatment conditions. It is known that p53, encoded by *tp53*, induces cell cycle arrest in response to endogenous and exogenous genotoxic damage [35]. However, reduced levels of cyclin D1, encoded by *ccnd1*, are required for DNA synthesis during the S phase in proliferating cells [36]. Therefore, the observed decrease in mRNA levels of p53 and cyclin D1 may indicate that embryonic cells overcome cell cycle arrest and undergo proliferation. However, the high expression of *check1*, *atm*, and *atr* in the CPT 24–48, Ola 2–24, and Ola 24–48 groups indicated that at least in some portions of embryonic cells checkpoint activity is increased. This assumption is supported by the low hatching rate in those groups.

The upregulation of several DNA repair pathways was observed in response to CPT and Ola exposure. Thus HR, BER, and NER were upregulated in embryos exposed to CPT at 24–48 hpf and to Ola at 2–24 and 24–48 hpf. A previous study showed that DSBs induced by Ola are repaired through the HR pathway in zebrafish embryos [28]. Similarly, CPT has been shown to induce DSBs repaired through HR in embryonic stem cells [37]. The activation of several DDR pathways in response to genotoxicity could be attributed to the cooperation between these pathways. Thus, the previous study showed the interaction between the HR and NER pathways in removing the inter-strand crosslinks (ICL) [38]. Furthermore, the interplay between BER and NER pathways is well documented [39]. Overall, in the developing sterlet embryos, it is possible that exposure to genotoxicants induced not only DSB but also SSB and ICL formation, as well as oxidative stress, which in turn required activation of several DDR pathways.

The exposure of sturgeon embryos to genotoxicants has affected the expression of genes associated with metabolism and the cell cycle. Transcriptomic changes were similar to the proteomic changes, in which the abundance of several metabolic proteins was modified. The association between DDR and metabolism is well known [40,41]. It has been previously shown that growth factors govern metabolism, while metabolism is a factor that drives the cell cycle and proliferation [42]. Furthermore, it is known that DNA damage causes cells to rewire their metabolism [41]. The obtained results suggest that DDR induces the adaptive response of metabolic pathways in developing fish embryos, which also affects the cell cycle and growth. In agreement with this conclusion, we also observed changes in vitellogenin (Vtg) abundance in embryos exposed to CPT and Ola (Supplementary Tables S2 and S3). In the oocyte, Vtg serves as one of the main sources of nutrients as well as free amino acids for protein synthesis during early embryogenesis [43]. A previous study reported changes in Vtg cleavage products in zebrafish embryos in response to toxic stress [44]. The stress response in sturgeon embryos altered the expression of genes associated with metabolism, and glycolysis, as well as the protein profiles of Vtg and glycolytic enzymes. Thus, DNA damage in sturgeon embryos is repaired at the cost of metabolic adjustments, changes in the cell cycle, and proliferation.

Another highly abundant protein in sturgeon embryos treated with genotoxicants compared with control embryos was zona pellucida (Zp) sperm-binding protein. In general, Zp proteins are expressed in egg chorion and provide thickness and hardness [45]. The study of [13] on the sterlet genome showed that there were 116 Zp genes in sterlet. The authors also noted that the possible reason for Zp gene family expansion in sturgeons is a protection mechanism against physical forces for the developing embryos. Based on the results of our study, it is possible to speculate that Zp proteins may also be involved in stress response or protect embryos against xenobiotic exposure. This may indicate the neofunctionalization of Zp proteins in sturgeon.

Although many similarities were observed in the embryo response to CPT and Ola, there were also compound-specific changes in the transcriptome and proteome of sturgeon embryos. For example, the analysis of protein profiles showed that treatment with Ola upregulated the expression of several proteins associated with oxidoreductase and antioxidant activities. These results agreed with previous studies showing that PARP-1 inhibition induced the formation of reactive oxygen species and oxidative DNA damage [46]. In contrast, exposure to CPT at 24–48 and 48–72 hpf affected proteins associated with metabolism, actin filaments, and cytoskeletal organization. A previous study has shown that CPT can interfere with microtubule polymerization and actin filaments during mitosis [47]. These side effects of CPT and Ola could explain the low Spearman’s correlation coefficients between DNA fragmentation and “omics” response (Table 2).

## 4. Materials and Methods

### 4.1. Ethics

Gametes were collected from live sterlets during the natural spawning period. Manipulations with broodstock were performed according to the law on the protection of animals against cruelty (Act no. 246/1992 Coll.) as a part of the sturgeon breeding program in the certified workplace (ref. number 6OZ3066/2020-18134, Czech Republic). Fish embryos were exposed to genotoxicants before reaching an independently feeding larval form. Therefore, no special approval from the local ethical committee was needed according to the laws to protect animals against maltreatment (Act no. 246/1992 Coll., Czech Republic).

### 4.2. Animals

As previously described [10], the sterlets (BW, 0.5–2 kg; 12 females, 12 males) were reared in the aquaculture facility of the Research Institute of Fish Culture and Hydrobiology at the University of South Bohemia, Vodňany, The Czech Republic. Before experimentation, fish were held in 5 m^3^ indoor tanks supplied with recirculating water system at 14–15 °C for 7 days prior to hormone stimulation. To induce ovulation, female fish were injected with carp pituitary extract powder (Rybníkářství Pohořelice, Czech Republic) dissolved in 0.9% (*w*/*v*) NaCl solution at an initial dose of 0.5 mg/kg of body weight. The second injection of carp pituitary powder (4.5 mg/kg of body weight) was performed 12 h after the first injection. Oocytes were collected 18–20 h after the second injection through a minimally invasive incision in the oviduct. Sperm production in males was induced by an intramuscular injection of carp pituitary extract powder (4.0 mg/kg of body weight) dissolved in 0.9% (*w*/*v*) NaCl solution. At 48 h post-injection, sperm was collected from the urogenital papilla with a catheter. The collected sperm was stored at 4 °C in separate cell culture containers (250 mL) until use. Eggs were fertilized with sperm (about 3 × 10^4^ spermatozoa per egg) activated in dechlorinated tap water at 15 °C for 2 min, and the stickiness of fertilized eggs was removed by three washes with 0.1% tannic acid (Sigma-Aldrich, St Louis, MO, USA) solution for 1 min. The identification of early developmental stages was based on studies of *Acipenser baerii* and *Acipenser güldenstädti* [48,49]. The embryos were incubated in Petri dishes with dechlorinated tap water at 16 °C; each Petri dish contained approximately 50 embryos.

### 4.3. Experimental Design

The compounds concentrations were selected based on the available literature data and our pilot experiments [10,26] (Supplementary Table S4). The final concentrations of 7 nM CPT (Sigma-Aldrich, St Louis, MO, USA, CAS Number: 7689-03-4) and 20 µM Ola (Selleck Chemicals, Houston, TX, USA) were used. Stock solutions 1000× concentrated (7 µM CPT and 20 mM Ola) were prepared in DMSO. Further, the stock solutions were added to the embryo culture medium (dechlorinated tap water) at a 1:1000 ratio to produce the required concentrations in the medium and 0.1% DMSO (*v*/*v*). The same amount of DMSO (0.1% in embryo culture medium) was used as a solvent control.

For each treatment, a group of 50 fertilized and developing embryos at the two-cell stage were transferred to a Petri dish. Each dish contained either vehicle control or a test compound in a total of 30 mL of dechlorinated tap water. Embryos were exposed to 7 nM CPT or 20 µM Ola for 24 h during one of three stages: (1) 2–24 h-post-fertilization (hpf) (blastula); (2) 24–48 hpf (gastrulation); (3) 48–72 hpf (neurulation). Genotoxicants were replaced with fresh water after 24 h exposure. Embryos were held in separate Petri dishes in dechlorinated tap water. Water was changed every day, and dead embryos were continuously removed. Six biological replicates were performed for each experiment.

Embryos were examined daily under a stereomicroscope (NSZ-608T, Nanjing Jiangnan Novel Optics Co., Ltd., Nanjing, China) and counted until hatching (8 days post-fertilization, dpf). The following parameters were analyzed:

- embryonic viability, % (number of live embryos/number of fertilized eggs × 100).
- hatching rate, % (number of hatched larvae/number of fertilized eggs × 100).
- occurrence of malformations.

The embryo phenotypes were observed under a microscope and imaged at 2.5× magnification. A minimum of 150 embryos were analyzed per treatment.

### 4.4. Comet Assay

DNA integrity was assessed using comet assays, or single cell gel electrophoresis assays, as described in our previous study [10]. Briefly, embryos at different stages were transferred into 1.5 mL tubes (3–4 embryos in each tube) with 1× PBS on ice. Embryos were mechanically disintegrated and centrifuged at 1000× *g* for 3 min. The pellets containing embryonic cells were washed with 1 mL of PBS, centrifuged at 1000× *g* for 3 min, and resuspended in 1 mL of PBS.

Microscope slides (OxiSelectST; Cell Biolabs, Inc., San Diego, CA, USA) used for the assay were prepared as follows: resuspended samples (200 µL) were mixed with 700 µL of 0.7% NuSieve GTG low melting point agarose (OxiSelectST; Cell Biolabs, Inc., San Diego, CA, USA). Forty microliters of this mixture was placed on the slide, and the agarose was allowed to solidify at 4 °C. Afterward, slides were immersed in lysis buffer (2.5 M NaCl, 100 mM EDTA, 10 mM Tris, 1% Triton X-100, 10% DMSO, pH 10) and incubated overnight at 4 °C. After the cells were lysed, the buffer was removed, and the slides were placed in a horizontal gel tank filled with freshly prepared electrophoresis buffer (90 mM Tris base, 90 mM boric acid, 2.5 mM EDTA). Electrophoresis was performed for 20 min at 35 V and 170 mA. Following electrophoresis, the slides were washed three times with pre-chilled DI H_2_O for 2 min. The washed slides were dehydrated with 70% ethanol, air-dried, and then stored at 4 °C. Before the analysis, 50 µL of PBS containing DAPI/Antifade Staining Solution (Sigma-Aldrich Co., St Louis, MO, USA) was added to the slides for agarose rehydration and DNA staining. The slides were analyzed using an Olympus B × 50 fluorescence microscope at 20× magnification. A minimum of 30 cells was scored for each sample. The images were analyzed with CometScore image analysis software (TriTek Corporation, USA). The percentage of DNA in the tail was calculated using the following formula: %Tail DNA = 100 × Tail DNA intensity/Cell DNA intensity

### 4.5. mRNA Extraction and Illumina Sequencing

The embryos for RNA sequencing were collected at 7 dpf and immediately transferred to a −80 °C freezer. Only embryos from the following groups were used for RNA sequencing: CPT 24–48, CPT 48–72, Ola 2–24, and Ola 24–48. Those groups were chosen based on the level of DNA damage and observed phenotypes. For each treatment, only embryos showing typical phenotypes were selected.

For total RNA extraction, 1 mL of TRI reagent (Sigma-Aldrich) was used in accordance with the manufacturer’s protocol and then re-precipitated with LiCl. Total RNA concentration was determined using a NanoDrop 2000 (Thermo Scientific, Waltham, MA, USA), and the quality of RNA was assessed using a Fragment Analyzer (Advanced Analytical). In total, three biological replicates (three embryos per condition) were prepared for sequencing.

### 4.6. RNAseq Data Analysis

Libraries for RNASeq were prepared using 700 ng of total RNA. The sequencing libraries were prepared using the SureSelect Strand-Specific RNA Library Prep for Illumina Multiplexed Sequencing (Agilent, G9691) based on the poly-A protocol. The libraries were sequenced on a NextSeq 500 instrument in PE75 high-output mode.

Approximately 5–10 million raw sequencing reads per sample were obtained. Adaptor sequences and low-quality reads were filtered using TrimmomaticPE (v. 0.36) with parameters “CROP:70 HEADCROP:12 ILLUMINACLIP: TruSeq-PE3.fa:2:30:10 LEADING:3 TRAILING:3 SLIDINGWINDOW:4:15 MINLEN:36” [50]. SortMeRNA (v. 2.1b) was used to remove any remaining rRNA and mtRNA reads [51]. The reads were pseudo-aligned to the transcriptome of *Acipenser baerii* (available online: http://publicsturgeon.sigenae.org/ngspipelines/#!/NGSpipelines/Acipenser%20baerii%20-%20publicSiberianSturgeon (accessed on 31 May 2022)) using kallisto (v. 0.43.1) and a count table generated. Before normalization, transcripts were removed that had zero counts in all samples.

Normalization of the raw counts and detection of the DEGs between the treatment and the control samples were determined using R (v. 4.1.0) software, DESeq2 (v. 1.32.0), using the median-of-ratios method, and a 0.1 *p*-adjusted (padj) cutoff [52]. Differentially upregulated and downregulated genes were defined as those with padj < 0.1 and log2foldchange greater than one or less than one respectively. Gene ontology (targeting biological processes) and pathway (KEGG) enrichment analysis was performed using gprofiler2 (v. 0.2.0) with the default parameters [53]. All the annotatable genes for *A. ruthenus* were used as a custom statistical background, with multiple testing correction performed using Set Counts and Sizes (g:SCS) with a significance cutoff of 0.05. Related Gene Ontology (GO) terms were then clustered and summarized using the simplifyEnrichment package (v. 1.2.0) with a *p*-adjusted threshold of 0.05, human reference annotation database, and the binary_cut clustering method [54]. Using Pathview (v. 1.32.0), KEGG maps were produced for the identification of the genes significantly upregulated and downregulated within the cell cycle (pathway: hsa04110), BER (pathway: hsa03410), mismatch repair (pathway hsa03430), NER (hsa03420), homologous repair (hsa03440) pathways [55]. Genes that were known to be involved in DNA damage repair, cell checkpoint, and cell cycle regulation ([56]; KEGG database) were subset from the RNASeq dataset and their fold-change and differential status relative to the control was queried.

The results of transcriptome analysis have been deposited in NCBI’s Gene Expression Omnibus and are accessible through GEO Series accession number GSE190524 (available online: https://www.ncbi.nlm.nih.gov/geo/query/acc.cgi?acc=GSE190524 (accessed on 9 December 2021)).

### 4.7. Protein Extraction and Two-Dimensional Gel Electrophoresis

Embryos at 7 dpf were mechanically dissociated in protein extraction buffer (8 M urea, 2 M thiourea, 4% CHAPS, 0.1% *w*/*v* Triton X-100, 100 mM dithiothreitol (DTT)) containing phosphatase inhibitors (1 mM sodium orthovanadate, 50 mM EDTA, 1 mM okadaic acid) and protease inhibitors (100 mM PMSF, 1 mg/mL pepstatin A, 5 mg/mL leupeptin). Proteins were extracted from at least four embryos per condition and analyzed separately. The analysis was repeated three times for each experimental condition. The Bradford assay was used to determine protein concentrations in each sample. The absorbance at 595 nm was measured using a Biobase-EL10A microplate reader (Biobase, Shandong, China).

The obtained protein mixture was used for two-dimensional gel electrophoresis (2D-PAGE). Isoelectric focusing was performed on ReadyStrip IPG strips (pH 3–10, 11 cm) with PROTEAN IEF (Bio-Rad, Hercules, CA, USA). In total, 150 µg of protein in 200 µL of rehydrating buffer (8M Urea, 2M Thiourea, 4% CHAPS, 50mM DTT, 0.4% IPG buffer) was applied to each IPG strip. The following conditions were used for separation: active rehydration at 50 V for 14 h; isoelectric focusing, 500 V for 1 h, 1000 V for 1 h, 3000 V for 1 h (gradient), and 8000 V for 2 h (gradient). After isoelectric focusing, the IPG strips were equilibrated in a buffer containing 6 M urea, 29.3% glycerol, 2% SDS, 75 mM Tris–HCl (pH 8.8), and 2% dithiothreitol for 15 min, followed by a second equilibration with a solution as above, but containing 2.5% iodacetamide instead of dithiothreitol, for a further 15 min. The strip was loaded on a 10% acrylamide gel for SDS-PAGE, and the proteins were separated by molecular weight using the Bio-Rad Mini-PROTEAN vertical electrophoresis system.

After electrophoresis, the gels were stained with Coomassie Brilliant Blue R-250 to visualize protein spots and scanned using the documentation system Fusion Solo 7S Edge (Vilber Lourmat, Collégien, France). The intensity of spots was analyzed using ImageJ (NIH, Bethesda, MD, USA). Only spots that were significantly changed between the control and treatment groups were selected for in-gel digestion and MS.

### 4.8. In-Gel Digestion and Protein Mass Spectrometry

After the gels were washed in water, the selected protein spots were cut from the gels. The gel pieces were destained by incubation with 50% *v*/*v* acetonitrile in 50 mM (NH_4_)HCO_3_ for 15 min. The gel pieces were treated with 5 µL of 50 mM (NH_4_)HCO_3_ containing 1 µg of modified sequencing-grade trypsin and incubated for 12 h at 37 °C. After digestion, the gel pieces were immersed in 100 µL of 0.1% *v*/*v* trifluoroacetic acid (TFA) in water for further peptide extraction. The peptides were concentrated and desalted using ZipTip pipette tips (Millipore, Billerica, MA, USA), which had been equilibrated by sequential washing with 100% acetonitrile, 50% acetonitrile–0.1% TFA, and 0.1% TFA in water. The sample was loaded on the ZipTip and contaminants were washed away with 0.1% TFA. Peptides were eluted with 2 µL of 50% acetonitrile–0.1% TFA in water. A 5 mg/mL solution of α-cyano-4-hydroxycinnamic acid (α-CHCA) in 50% acetonitrile–0.1% TFA was used as the matrix. One microliter of each sample was mixed with 1 µL of freshly made CHCA matrix and spotted on a steel MALDI target plate (MT 34 Target Plate Ground Steel, Bruker Daltonics, Billerica, MA, USA). The peptide samples were analyzed using a time-of-flight Autoflex-TOF/TOF mass spectrometer (MALDI-TOF-TOF, Bruker Daltonics, Bremen, Germany). The MS and MS/MS LIFT spectra of selected ions were collected and calibrated externally using monoisotopic [M+H]+ ion peptide calibration standards (Bruker Daltonics, Billerica, MA, USA) consisting of angiotensin II (1046.54), angiotensin I (1296.68), substance P (1347.73), bombesin (1619.82), ACTH clip 1 (2093.086), ACTH clip 18 (2465.19), and somatostatin 28 (3147.471). The MS peptide mass fingerprint (PMF) and fragment mass spectra (MS/MS) from each individual spot were combined and searched against the Acipenseriformes proteins (155374 residues) deposited in the NCBIr database (version from 2 February 2021) using MascotServer (Matrix Sciences, Chicago, IL, USA) with the following settings: cleavage enzyme, trypsin; max missed cleavages, 2; mass tolerance mono, 50 ppm; fragment ion mass tolerance, 0.5 Da; parent ion mass tolerance, 100 ppm; alkylation of cysteine by carbamidomethylation as a fixed modification; and oxidation of methionine as a variable modification. For the PMF and MS/MS ion search, statistically significant (*p* ≤ 0.05) matches by MASCOT were regarded as correct hits.

## 5. Conclusions

This study is the first report on transcriptomic and proteomic changes induced by DNA damage in sturgeon embryos. It was revealed that several DNA repair pathways were activated (HR, BER, NER, MMR) and DNA repair was closely associated with metabolic changes (oxidative phosphorylation, sugar metabolism, etc.). Thus, apart from DNA damage, genotoxicant exposure can be associated with oxidative stress, changes in metabolism, and affected organ development. The effect of genotoxicants and the level of DNA damage were stage-specific with prior-neurulation stages being more sensitive.

Our results highlighted the correlation between phenotype formation and the “omics” response. Treatments that induced DNA damage and phenotype formation were associated with changes in expression of the DDR genes: *check1, atm, atr, xpc*, *rad50*, *xpa*, *xrcc1*, *msh2*, *rpa1*, *ercc5*, *pold3*, *ercc2*, *fen1*, *blm*, *rad51ap1*, *nbn*, and *eme1.* The presented pieces of evidence suggest that these genes could be used as markers of DNA damage and DDR. In contrast, several genes were modified irrelevant of phenotype formation: *tfdp2*, *tp53bp1*, *ddb1*, *parp8*, *apex1*, *rad52*, *ogg1, ccnd1*, *tp53*, *parp9*, and *xrcc5*. It can be concluded that the second group of genes is related to the adaptive stress response. We found that the sturgeon DDR mechanisms have some degree of similarity with previously reported zebrafish DDR, which agrees with the high level of DDR evolutionary conservation. Thus, it could be speculated that the obtained markers could find their use in toxicological studies on a broad variety of fish species.

Although DDR mechanisms were mainly conserved in sturgeons, we have observed a species-specific response to genotoxicant exposure, namely overexpression of Zp proteins. This result may indicate the neofunctionalization of Zp proteins in sturgeon. Further studies are needed to investigate possible long-lasting effects of stress in sturgeon embryos and the protective role of Zp protein. Finally, this study provides new insights into embryo susceptibility to DNA damage in endangered sturgeons, which is particularly important for sustainable ex situ conservation efforts.

## Figures and Tables

**Table 1:** Summary of the results obtained in the current study for sterlet (*A. ruthenus*) embryos exposed to 7 nM camptothecin (CPT) and 20 µM olaparib (Ola) at different stages of embryo development: 2–24, 24–48, and 48–72 h post-fertilization. The maximum value of DNA fragmentation is shown for each condition. Viability and hatching rate are shown for 7 days post-fertilization.

Parameter	Pathway	Treatment Groups					
		CPT 2–24	CPT 24–48	CPT 48–72	Ola 2–24	Ola 24–48	Ola 48–72
**Viability**		0%	31%	47.6%	40.2%	42%	45.2%
**Hatching rate**		0%	2%	40.9%	6.8%	32.8%	40.4%
**DNA fragmentation (max)**		21.3%	14.2%	4.6%	9.8%	14.1%	5.2%
**RNASeq**			√	√	√	√	
Upregulated KEGG	Wnt signaling		√	√	√	√	
	MAPK signaling		√	√	√	√	
	Notch signaling		√	√	√	√	
	Nucleotide excision repair		√		√	√	
	Homologous recombination		√		√	√	
	Base excision repair		√		√	√	
	Mismatch repair				√	√	
	mTOR signaling pathway					√	
	Autophagy					√	
Downregulated KEGG	Oxidative phosphorylation		√	√	√	√	
	Autophagy		√		√		
**Proteomics**			√	√	√	√	
Upregulated KEGG	Membrane trafficking		√	√	√	√	
	Metabolic pathways				√	√	
	Peroxisome				√	√	
Downregulated KEGG	Cytoskeleton proteins		√	√			
	Regulation of actin cytoskeleton		√	√			

In the Ola-treated group, 15 DAPs were identified. Similar to the CPT treatment group, tubulin beta chain-like isoform X3, alpha-enolase isoform X2, and creatine kinase M-type were downregulated (Supplementary Table S3). One protein was upregulated in the same way as its corresponding mRNA, namely protein disulfide-isomerase A3. In contrast to CPT, embryos treated with Ola had a higher number of upregulated proteins involved in oxidoreductase and antioxidant activity.

**Table 2:** Correlation between parameters analyzed in the current study on sterlet (*A. ruthenus*) embryos. The correlation coefficients between % viability, hatching rate, DNA fragmentation, number of differentially expressed genes (DEGs), and number of differentially abundant proteins (DAPs) were measured by Spearman’s test. The *t*-test is used to establish the significance of the correlation between pairs of parameters. Asterisks indicate *p* < 0.05.

	Viability	Hatching	DNA Fragmentation	DEGs	DAPs
Viability	1.00	**0.95** *	−0.73	**−0.91** *	**−0.88** *
Hatching	**0.95** *	1.00	−0.62	**−0.93** *	−0.87
DNA fragmentation	−0.73	-0.62	1.00	0.73	0.38
DEGs	**−0.91** *	**−0.93** *	0.73	1.00	0.68
DAPs	**−0.88** *	−0.87	0.38	0.68	1.00

## Data Availability

The data discussed in this publication have been deposited in NCBI’s Gene Expression Omnibus and are accessible through GEO Series accession number GSE190524 (available online: https://www.ncbi.nlm.nih.gov/geo/query/acc.cgi?acc=GSE190524 (accessed on 9 December 2021)).

## Supplementary Materials

The following supporting information can be downloaded at: https://www.mdpi.com/article/10.3390/ijms23126392/s1.
