# Supplementary material for: Transcriptome and Proteome Analyses Reveal Stage-Specific DNA Damage Response in Embryos of Sturgeon (Acipenser ruthenus)
Source: Int J Mol Sci. 2022 Jun 7;23(12):6392. doi: 10.3390/ijms23126392 (PMC9223696; doi:10.3390/ijms23126392)
Supplement: Supplementary file 1 [file ijms-23-06392-s001.zip › Supplementary figures - 12102021.pdf]

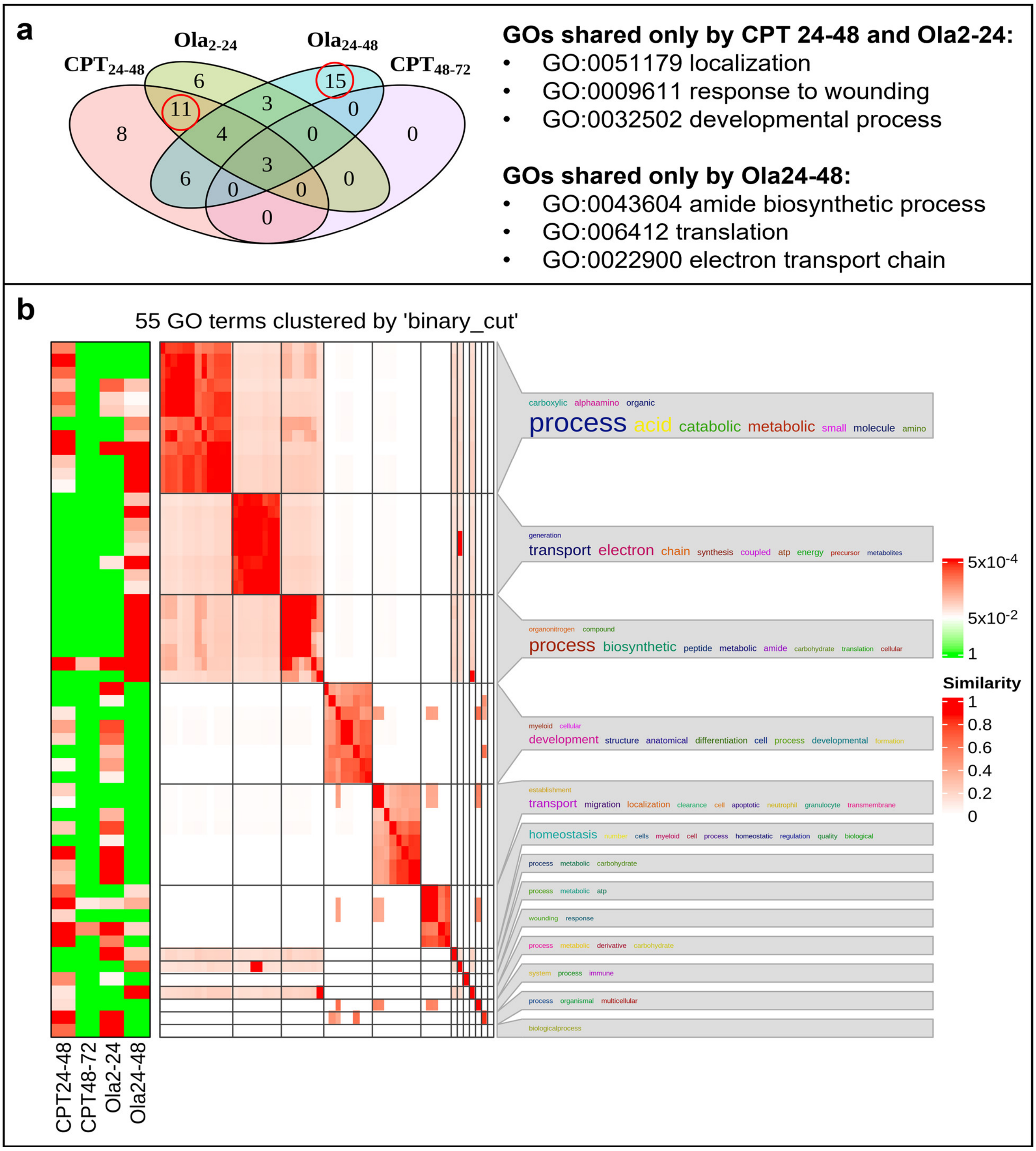

Supplementary Figure S1. Summary of significantly (padj < 0.05) enrichment Gene Ontology (GO) terms associated with the down-regulated differentially expressed genes. (**a**) Overlap of the enriched GO terms between each treatment condition. Shown is a subset of the list of Biological Processes shared by some of the treatment groups. (**b**) Summarized GO terms. Left most heatmap represents the padj values for the enriched GO terms. Second heatmap shows the similarity matrix of the GO terms.

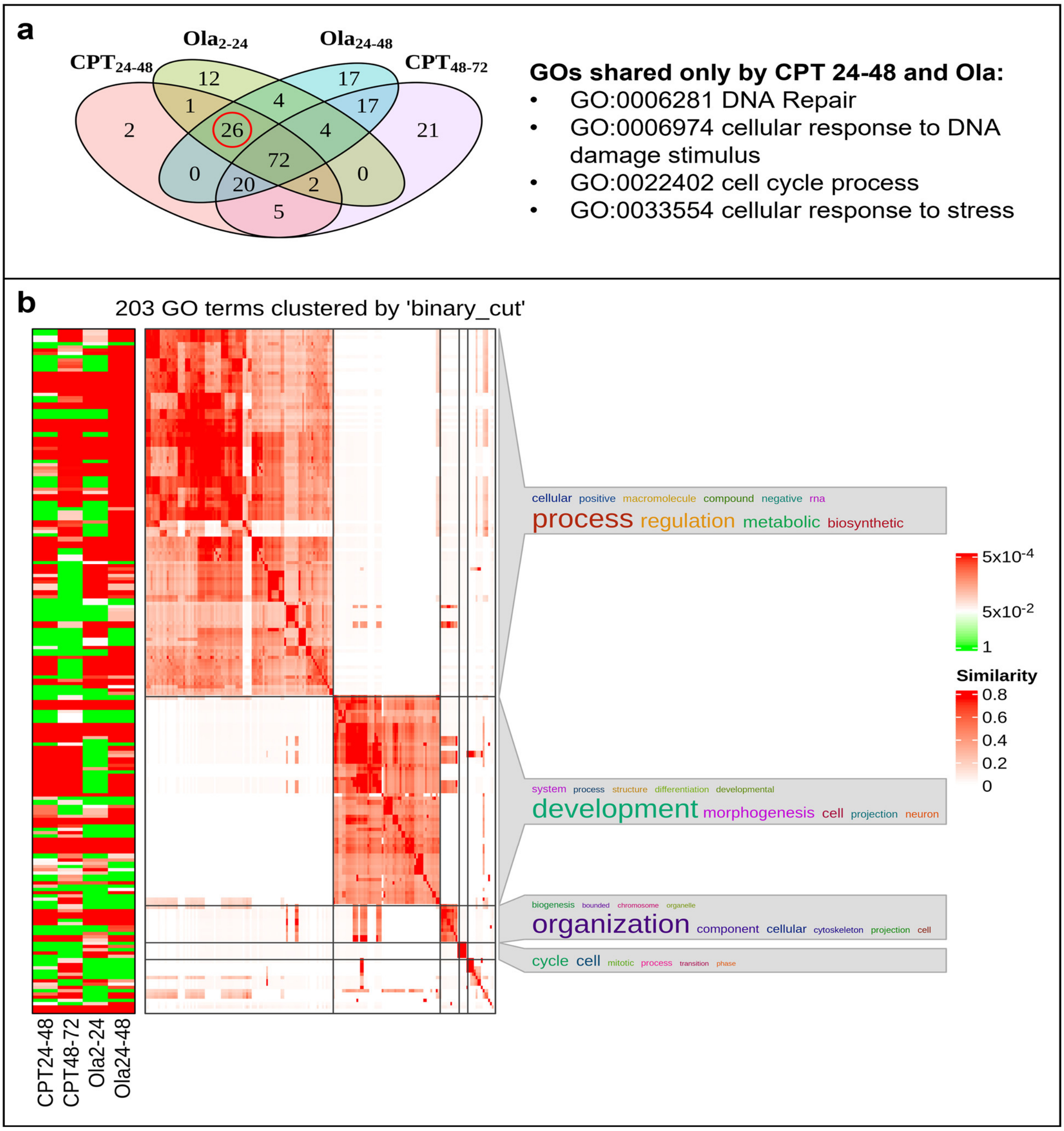

Supplementary Figure S2. Summary of significantly (padj < 0.05) enrichment Gene Ontology (GO) terms associated with the up-regulated differentially expressed genes. (**a**) Overlap of the enriched GO terms between each treatment condition. Shown is a subset of the list of Biological Processes shared by CPT 24-48 and the Ola treatment groups related to DNA damage response. (**b**) Summarized GO terms. Left most heatmap represents the padj values for the enriched GO terms. Second heatmap shows the similarity matrix of the GO terms.

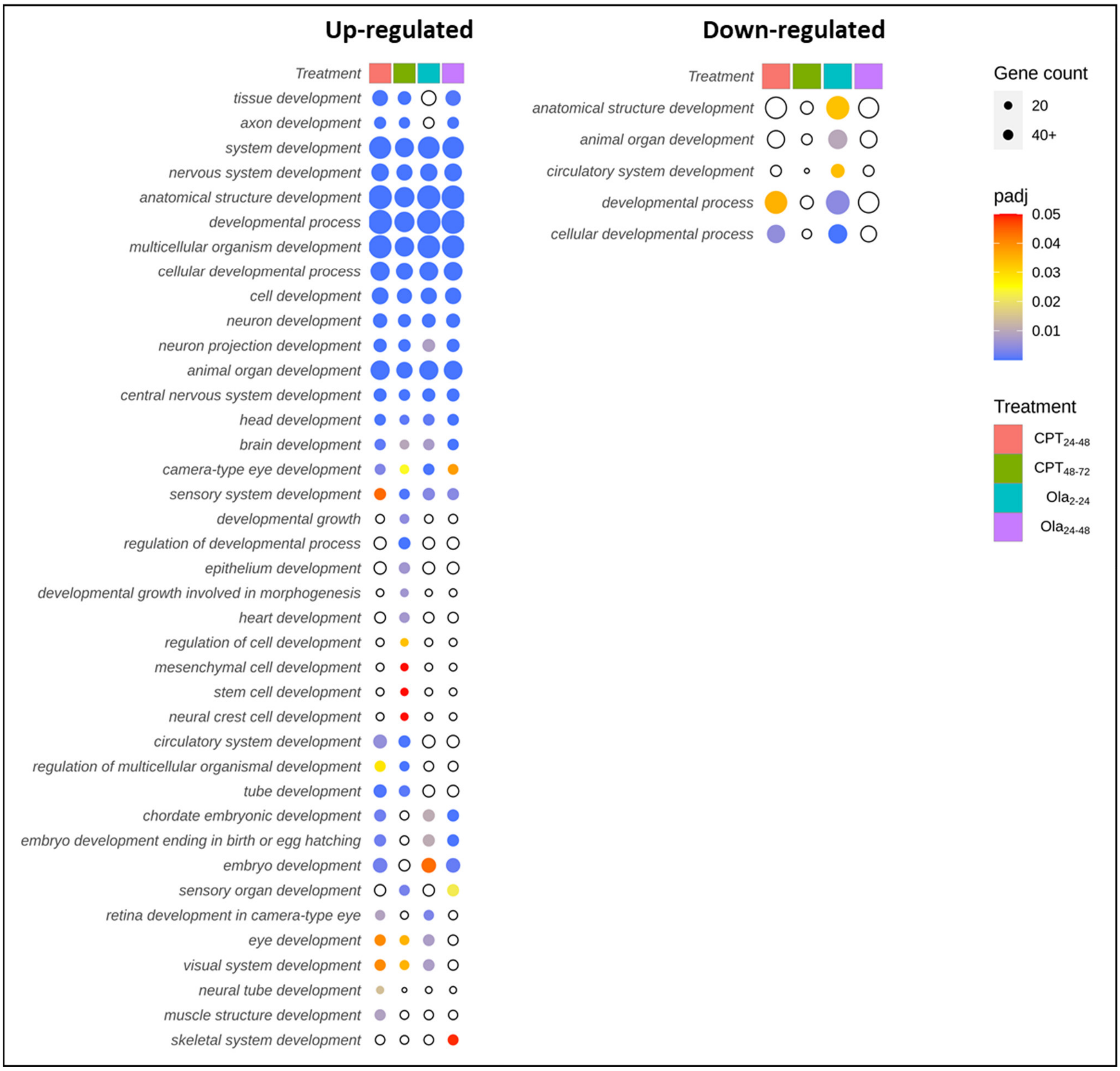

Supplementary Figure S3. List of significantly enriched Gene Ontology terms associated with Developmental Biological Processes that were either up-regulated or down-regulated within the treated samples versus the control. Color scale represents the adjust p-values less than 0.05, with empty circles showing those greater than 0.05. The size of the circle represents the number of significantly differentially expressed genes that are part of the given GO term

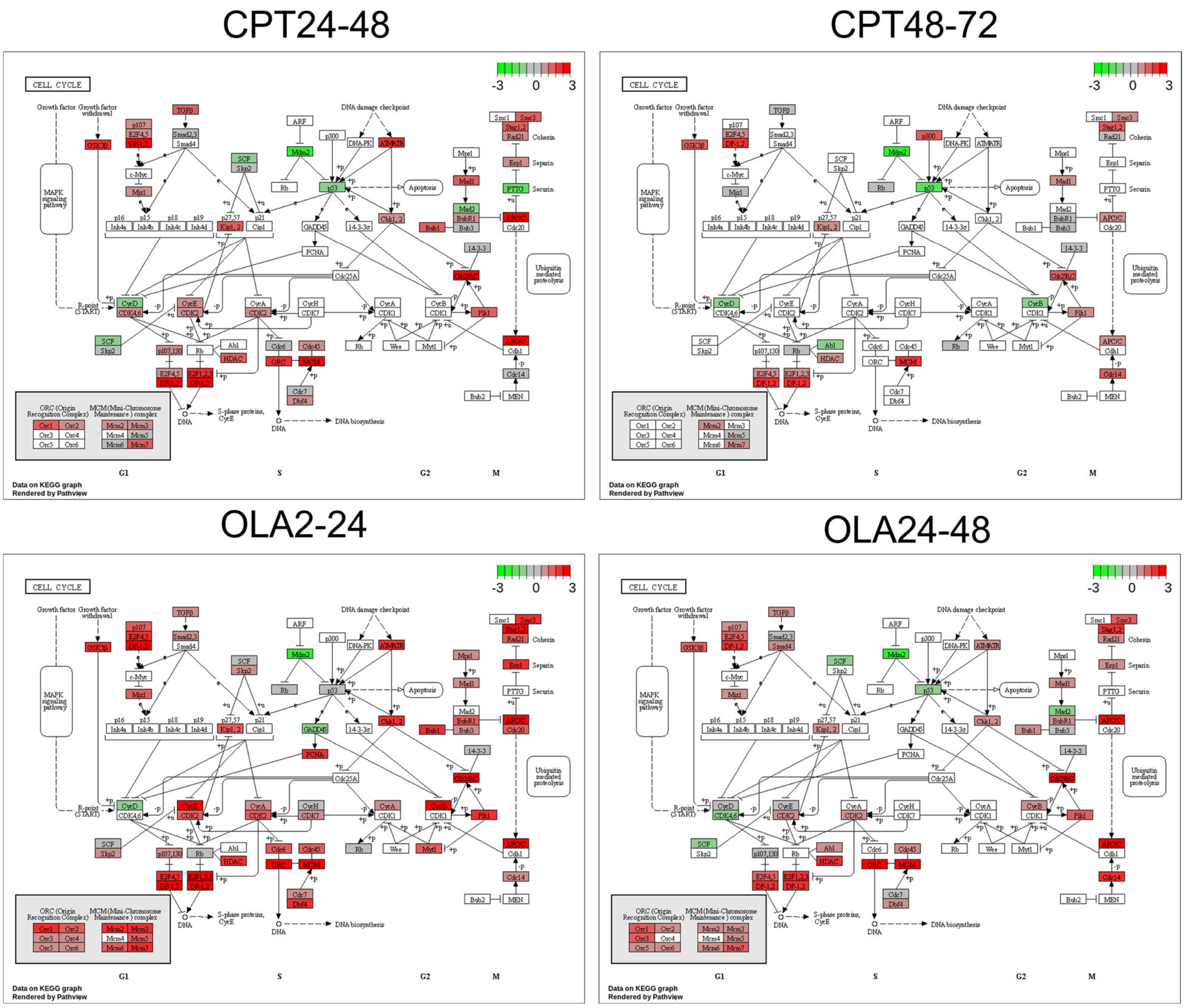

Supplementary Figure S4. Significantly (padj < 0.1) differentially expressed genes mapped to the KEGG pathway, cell cycle (hsa04110). Color scale represents the Log2 fold change between the treated and the control samples, capped at a maximum of 3 and minimum of -3.

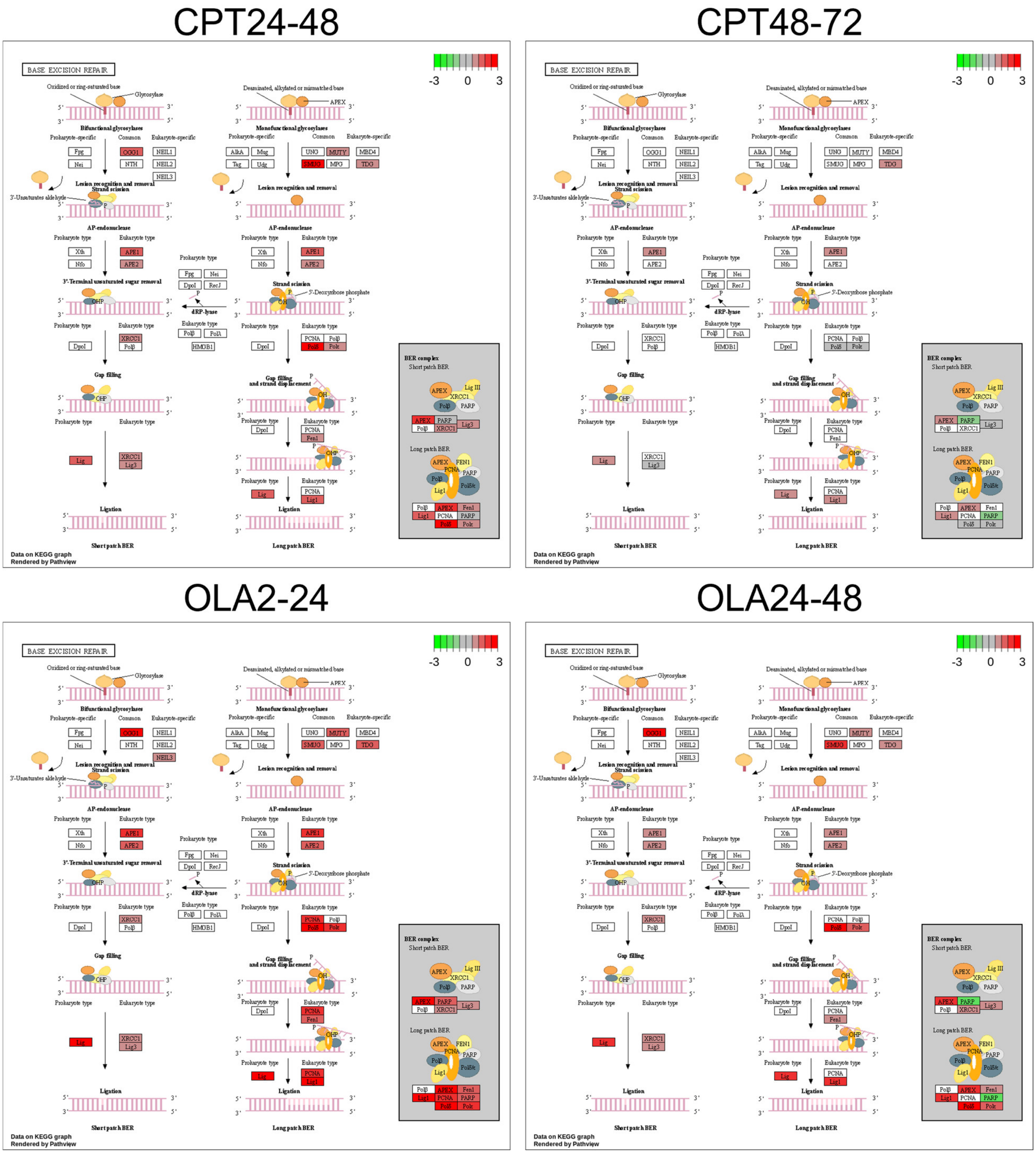

Supplementary Figure S5. Significantly (padj < 0.1) differentially expressed genes mapped to the KEGG pathway, base excision repair (hsa03410). Color scale represents the Log2 fold change between the treated and the control samples, capped at a maximum of 3 and minimum of -3.

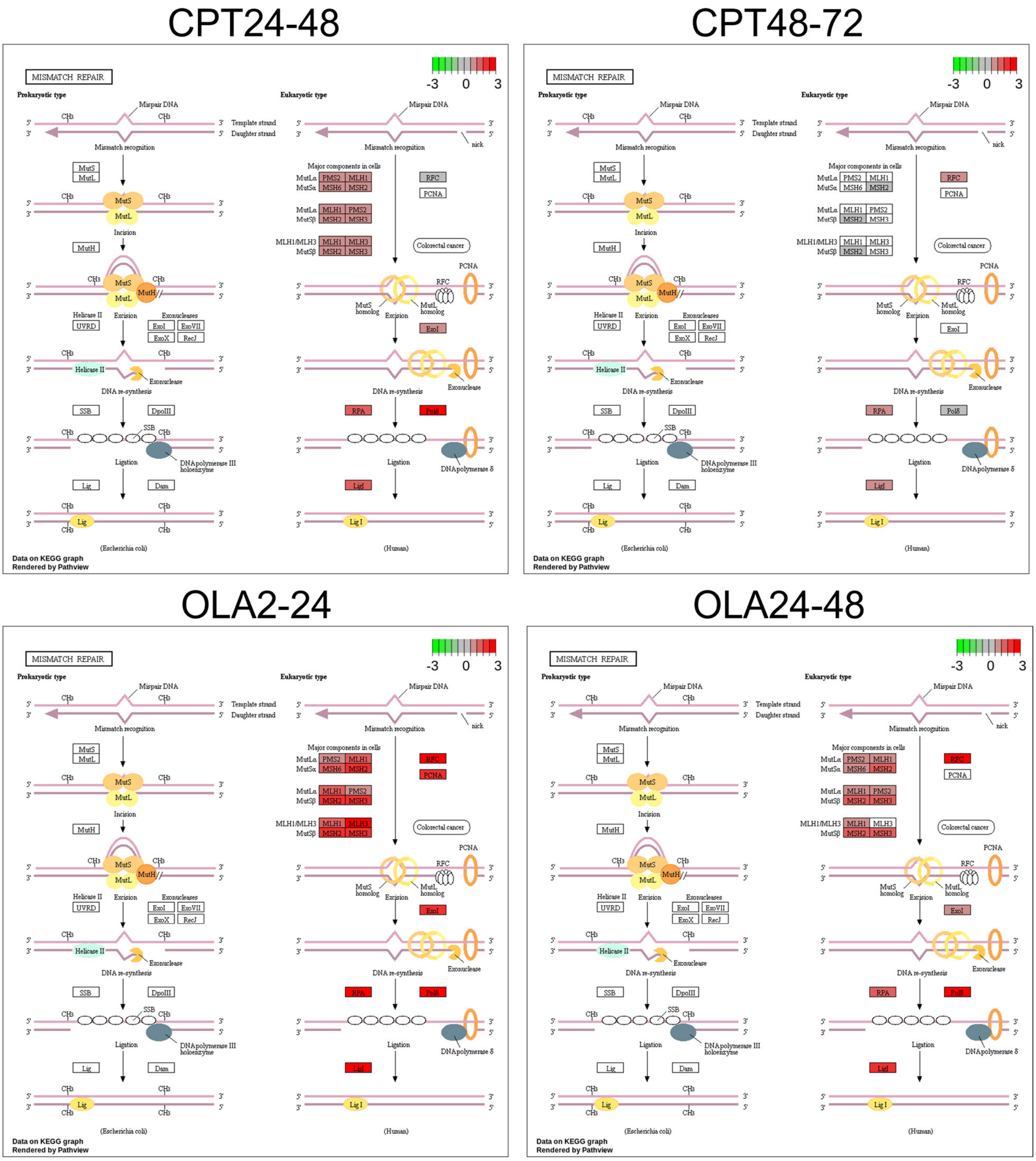

Supplementary Figure S6. Significantly (padj < 0.1) differentially expressed genes mapped to the KEGG pathway, mismatch repair (hsa03430). Color scale represents the Log2 fold change between the treated and the control samples, capped at a maximum of 3 and minimum of -3.

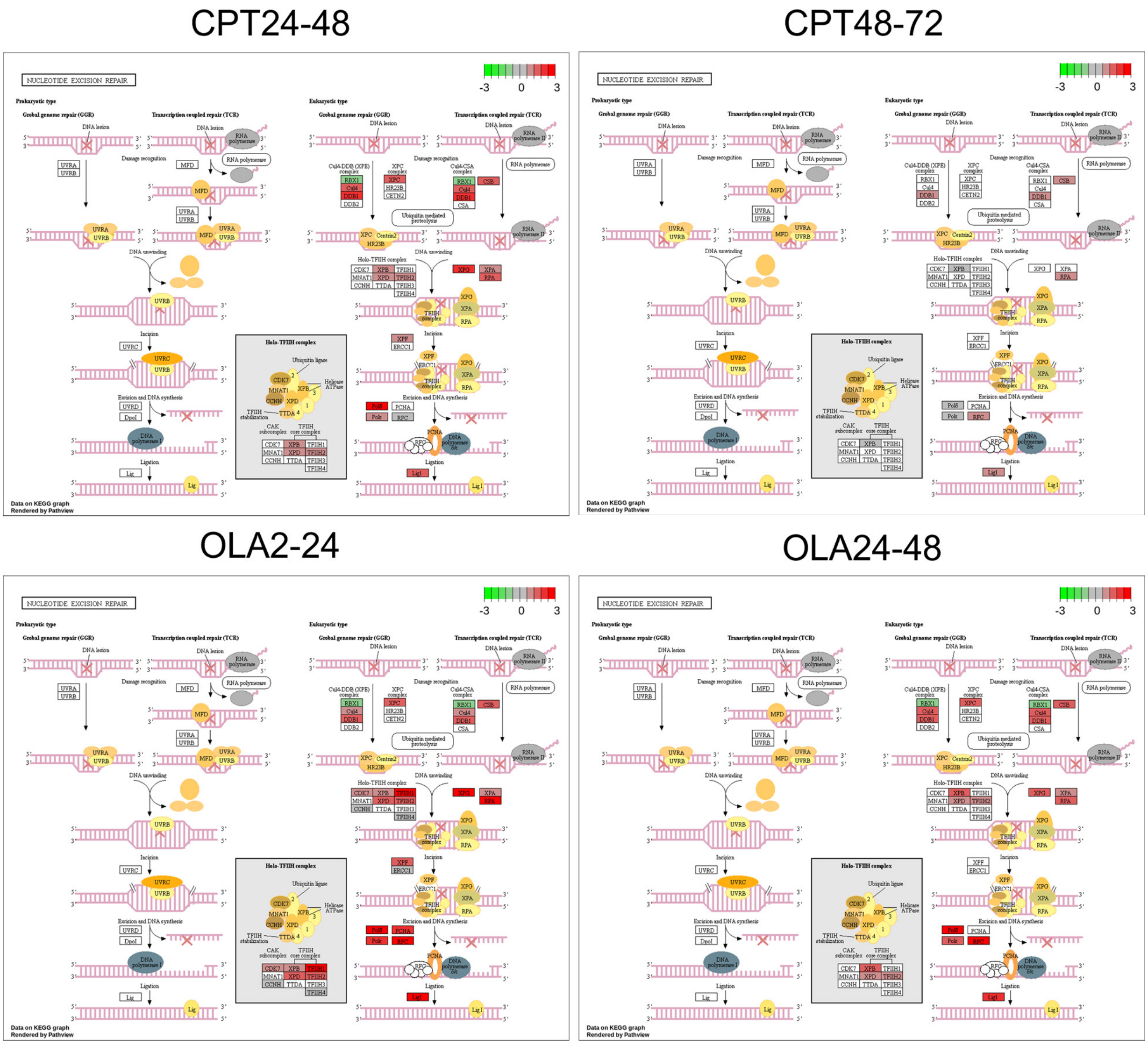

Supplementary Figure S7. Significantly (padj < 0.1) differentially expressed genes mapped to the KEGG pathway, nucleotide excision repair (hsa03420). Color scale represents the Log2 fold change between the treated and the control samples, capped at a maximum of 3 and minimum of -3.

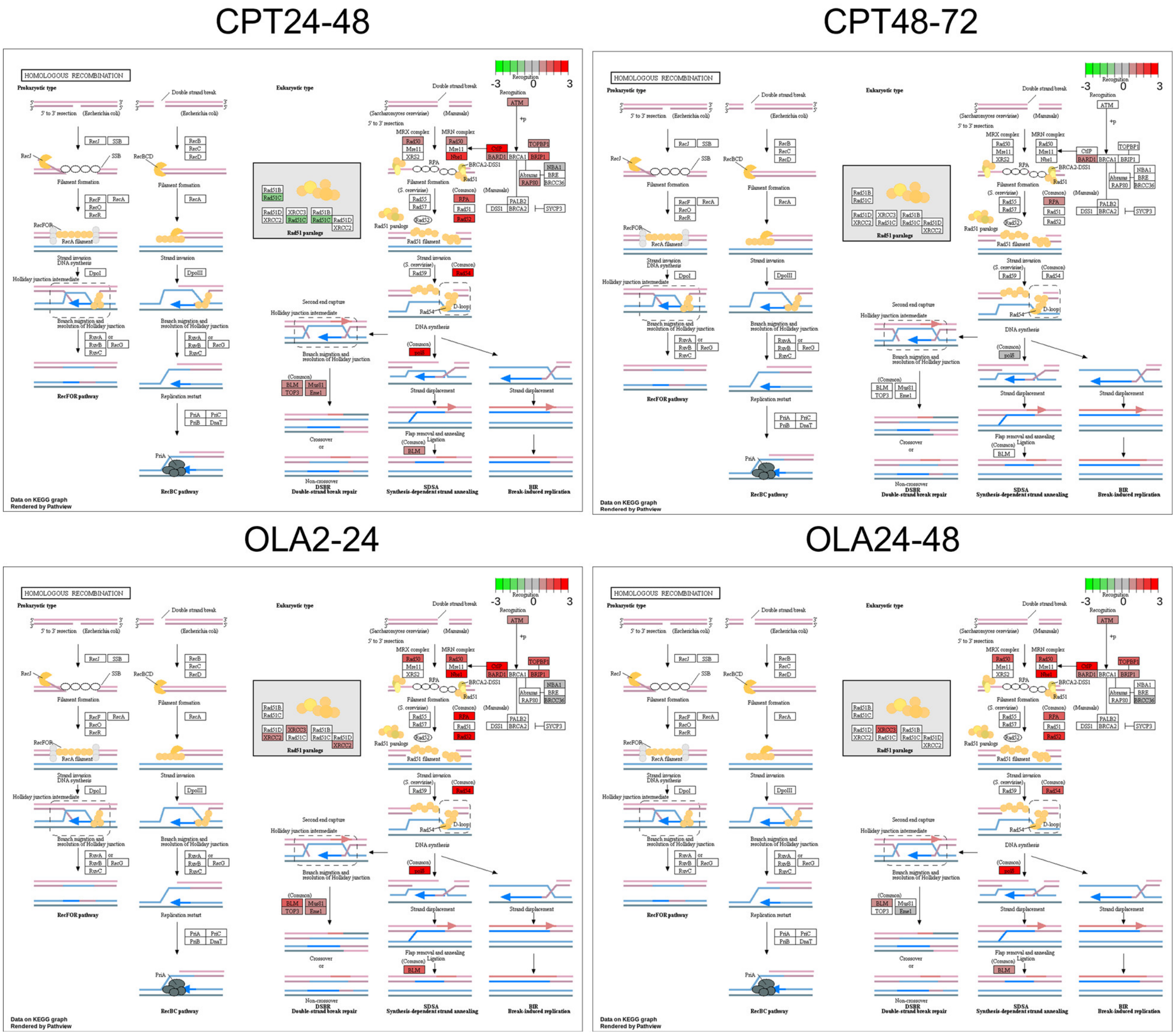

Supplementary Figure S8. Significantly (padj < 0.1) differentially expressed genes mapped to the KEGG pathway, homologous recombination (hsa03440). Color scale represents the Log2 fold change between the treated and the control samples, capped at a maximum of 3 and minimum of -3.

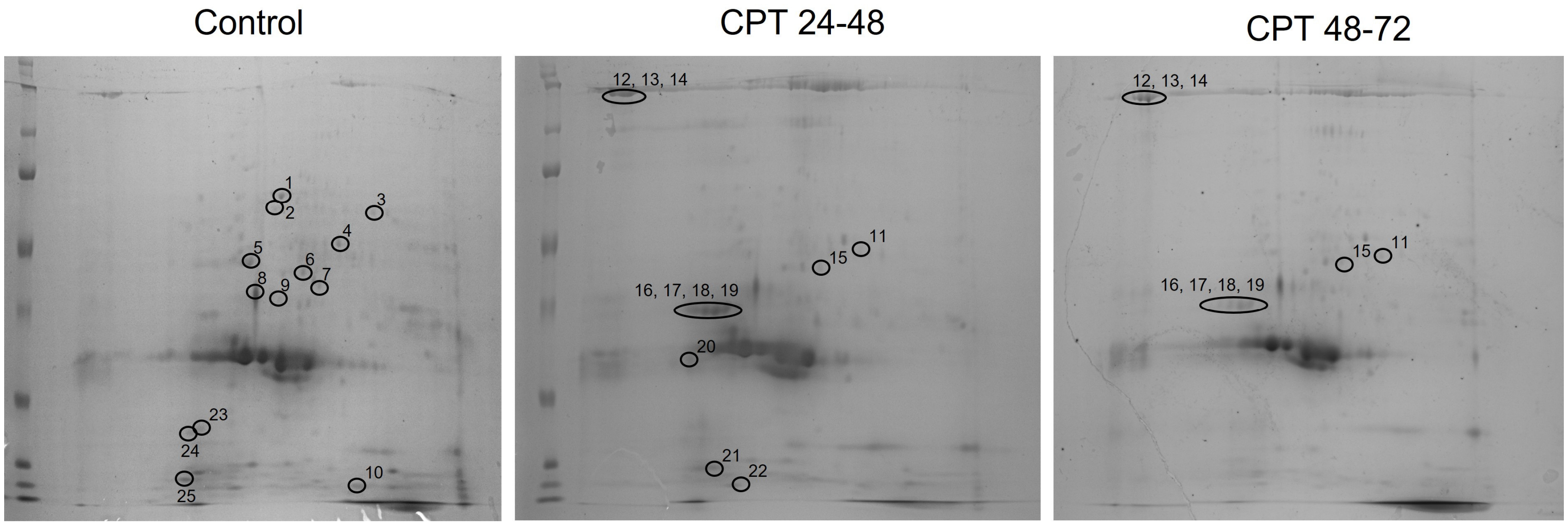

Supplementary Figure S9. Two-dimensional gel electrophoresis of sterlet embryo proteins following exposure to CPT. The separated proteins were stained with Coomassie Brilliant Blue R-250.

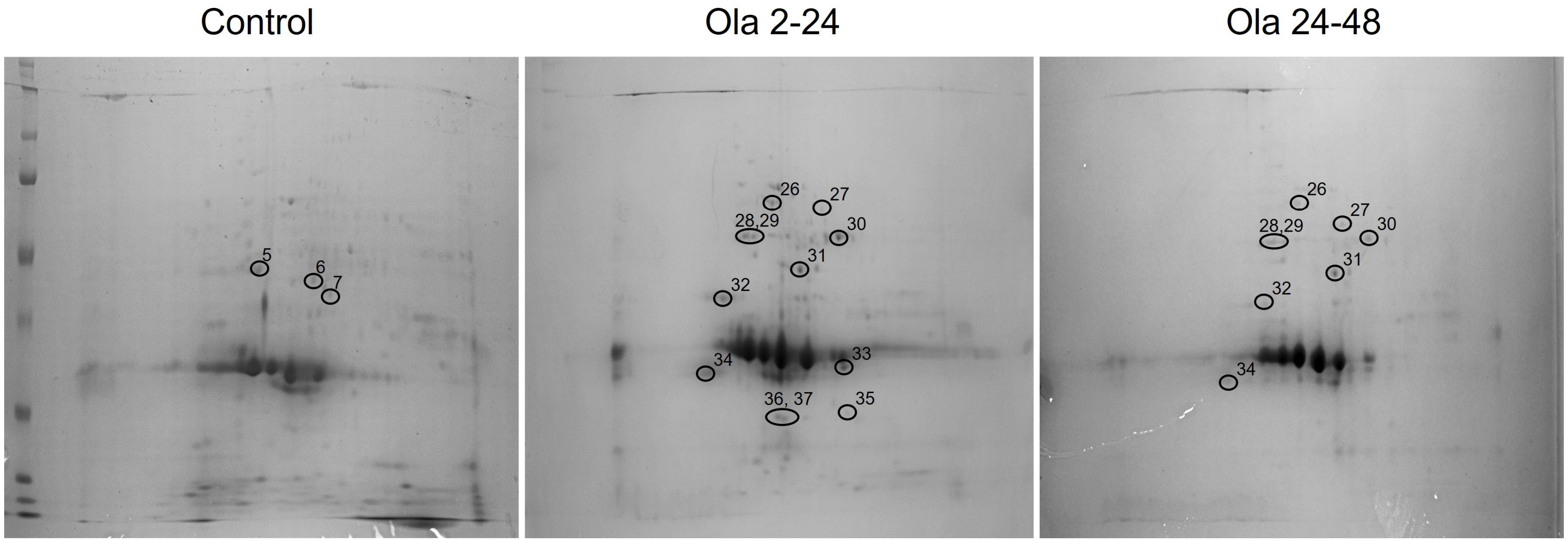

Supplementary Figure S10. Two-dimensional gel electrophoresis of sterlet embryo proteins following exposure to Olaparib. The separated proteins were stained with Coomassie Brilliant Blue R-250.
