# Supplementary material for: Transcriptome and Proteome Analyses Reveal Stage-Specific DNA Damage Response in Embryos of Sturgeon (Acipenser ruthenus)
Source: Int J Mol Sci. 2022 Jun 7;23(12):6392. doi: 10.3390/ijms23126392 (PMC9223696; doi:10.3390/ijms23126392)
Supplement: Supplementary file 1 [file ijms-23-06392-s001.zip › Supplementary Table S4.pdf]

Supplementary Table S4. Sterlet embryo viability and hatching rate following exposure to different concentrations of olaparib (5, 10, 20 μM). Embryos were exposed to olaparib from 2 till 24 hours post-fertilization. All results are presented at 8 days post-fertilization. Results represent mean of two independent experiments, number of embryos N = 100.

Treatment	Live,%	Hatched,%
Control	91	76
Olaparib 5 μM	96	62
Olaparib 10 μM	92	56
Olaparib 20 μM	40	7
